# Advanced cell therapeutics are changing the clinical landscape: will mesenchymal stromal cells be a part of it?

**DOI:** 10.1186/s12916-019-1289-6

**Published:** 2019-03-05

**Authors:** Richard Schäfer

**Affiliations:** 1 Institute for Transfusion Medicine and Immunohaematology, German Red Cross Blood Donor Service Baden-Württemberg-Hessen gGmbH, Goethe University Hospital, Sandhofstrasse 1, 60528 Frankfurt am Main, Germany; 2 Center for Stem Cell Biology and Regenerative Medicine, University of Maryland School of Medicine, 655 W Baltimore St, BRB 14-021, Baltimore, MD 21201 USA

**Keywords:** Cell therapy, Stem cells, Mesenchymal stromal cells, Immunotherapy, Regenerative medicine, Biotechnology

## Abstract

During the past 15 years there have been dramatic changes in the medical landscape, particularly in oncology and regenerative medicine. Cell therapies have played a substantial part in this progress. Cellular immunotherapies can use immune cells, such as T cells or natural killer cells that, after functional modification ex vivo, exert powerful anti-cancer effects when given to the patient. Innovative technologies, such as re-programming terminally differentiated cells into pluripotent stem cells or into other cell types and applying specific enzymes to more precisely edit the human genome, are paving the way towards more potent cell and gene therapies.

Mesenchymal stromal cells are promising cellular immunotherapeutics, which also have potential for use in tissue engineering strategies and other regenerative medicine applications. However, substantial gaps in our knowledge of their biology and therapeutic efficacy present major challenges to their sustainable implementation in the clinical routine.

In this article, progress in the field of cell therapeutics during the past 15 years will be briefly discussed, with a focus on mesenchymal stromal cells, highlighting the impact of this field on patient care.

## Background

When BMC Medicine was inaugurated 15 years ago, available cell therapies were mainly haematopoietic stem cell transplantations, which had been established as standard treatment for haematologic malignancies. However, allogeneic haematopoietic stem cell transplantation carried a major risk of developing life-threatening complications, such as non-engraftment, serious infections and graft-versus-host disease (GvHD) [1]. Within this period, groundbreaking novel technologies were also developed; for example, re-programming of differentiated cells into induced pluripotent stem cells (iPSC) [2, 3] and precise enzymatic genome editing [4], both providing yet unknown options for cell and gene therapies. Advancing adoptive cellular immunotherapy, novel insights into interactions between immune cells and cancerous tissue, efficacious cell collection using optimised apheresis techniques, as well as sophisticated ex vivo-cell engineering, enabled the introduction of chimeric antigen receptor (-T) cell therapies into the clinic [5]. Personalised vaccination strategies use patient-derived cancer cells to generate individual dendritic cell-based vaccines that were successfully applied against malignancies including ovarian cancer and acute leukaemia [6, 7].

Based on findings of the therapeutic potential of non-haematopoietic precursor cells [8, 9], early experimental cell therapy concepts had been suggested to regenerate damaged tissue, particularly the heart [10–12], heralding the field of regenerative medicine at the beginning of the new millennium. Because of their immunomodulatory and regenerative effects, mesenchymal stromal cells (MSCs) were extensively evaluated for their potential uses in cellular immunotherapy and regenerative medicine. MSCs can be isolated from a variety of tissues such as bone marrow (BM), adipose tissue, cord (blood), or amniotic fluid [13], as well as from iPSC, with the potential of an inexhaustible source [14]. Here I elaborate on significant developments in MSC therapies during the past 15 years.

## Immunomodulation cell therapies

Interacting with different immune cell subsets, MSCs exert immunomodulatory effects in vitro, such as suppressing activated T cell proliferation and cytokine production. They have been shown to induce a tolerogenic immune phenotype in vivo, as characterised by a decrease in pro-inflammatory IL-17 positive T cells and an increase in regulatory T cells [13, 15, 16]. These observations suggest that MSCs may be interesting candidates for the treatment of immunopathologies. Indeed, MSC therapeutics have been applied in multiple clinical trials for GvHD and organ graft rejection, as well as for autoimmune diseases like multiple sclerosis, myasthenia gravis or type 1 diabetes mellitus [16, 17]. Yet, clinical results over the past decade have been variable [16]. Specifically, an allogeneic MSC product for GvHD therapy performed disappointingly in 2009 [18], dampening initial enthusiasm. Also, a recent Cochrane review of numerous clinical trials [19] found insufficient evidence that MSCs were an effective therapy for GvHD. Innovative approaches for MSC-mediated GvHD therapy include MSC-derived extracellular vesicles [20]; pooling of BM-derived mononuclear cells to generate a more standardised MSC product with robust immunomodulation capacity [21]; and measuring the ability of immune cells to kill MSC, thereby identifying patients who respond to MSC immunotherapy [22].

## Regenerative medicine

MSCs, without or with genetic modifications or other *ex vivo* manipulations to increase their therapeutic potential, have been shown to exert therapeutic effects in diseases of various organs, including the heart, lung, liver, pancreas, kidney, skeletal system and the central nervous system [23, 24]. To date, MSCs have been assessed for regenerative applications in numerous clinical trials, with the main sources being BM and adipose tissue [24]. As MSCs feature the potential for mesodermal differentiation in vitro, direct tissue replacement of damaged tissue by differentiated MSCs was initially postulated as a mechanism of action [25]. However, growing evidence has shifted towards paracrine factors and extracellular vesicles being responsible for mediating immunomodulatory and regenerative MSC functions [23, 26]. Novel technologies allow the large-scale production of MSCs in bioreactors [27]; MSC can also be applied, with or without scaffolds, in tissue engineering concepts [28] for disease modelling and therapy.

## Challenges and novel approaches

The past decade has shown that, despite encouraging clinical data, major challenges prevail before MSC therapies can be sustainably implemented in the clinical routine. To date, the poorly understood heterogeneity of MSCs means that major issues are yet to be addressed; for example, between individuals and within respective MSC preparations, variable manufacture technologies, and minimally defined media supplements (such as fetal calf serum or human platelet lysate) [29, 30]. Consequently, it is difficult to compare MSC therapeutics because they lack standardized quality and there are only few measures available – some of debatable relevance – to assess their potency. Therefore, it remains unclear as to which patients will ultimately profit from these therapies.

Advanced technologies, like single cell analyses, give deeper insights into MSC heterogeneity, allowing functional cell clusters and/or molecular signatures to be identified, which could be linked to their therapeutic potential [31, 32].

## Conclusions

During the past 15 years, technological hallmarks like iPSC generation, genome editing and single cell analysis platforms have been developed. This biotechnological progress has led to significant achievements in the cell therapy field, including MSC-mediated immunomodulation and tissue regeneration. This progress is encouraging and the clinical MSC field is, after some stagnation, now regaining momentum.

Better understanding MSC heterogeneity, their mechanisms of action and evidence-based identification of patient cohorts who might benefit from MSC therapeutics, could help to sustainably translate these therapies to the clinic.

## Abbreviations

BM: Bone marrow
GvHD: Graft-versus-host disease
iPSC: Induced pluripotent stem cells
MSC: Mesenchymal stromal cells
